# Supplementary material for: Invasive Pneumococcal Diseases in People over 65 in Veneto Region Surveillance
Source: Vaccines (Basel). 2024 Oct 23;12(11):1202. doi: 10.3390/vaccines12111202 (PMC11598215; doi:10.3390/vaccines12111202)
Supplement: Supplementary file 1 [file vaccines-12-01202-s001.zip › vaccines-3262834-SI.pdf]

## Supplementary materials

**Table S1.** Joinpoint regression analysis results of notification rates expressed as annual percentage change (APC) and average APC (AAPC).

| Variable | Segment | Lower Endpoint | Upper Endpoint | APC | 95% CI | P-Value |
|---|---|---|---|---|---|---|
| **Overall** | 1 | 2007 | 2018 | 6.5802* | (3.41; 11.57) | < 0.000001 |
| | 2 | 2018 | 2021 | -35.0133* | (-43.38; -20) | < 0.000001 |
| | 3 | 2021 | 2023 | 125.4183* | (65.77; 193.81) | < 0.000001 |
| | Average (AAPC) | 2007 | 2023 | 6.6733* | (3.51; 9.78) | < 0.000001 |
| **Age 65 -74** | 1 | 2007 | 2018 | 6.0920* | (2.6; 12.72) | 0.003599 |
| | 2 | 2018 | 2021 | -32.6622* | (-42.93; -15.61) | 0.002 |
| | 3 | 2021 | 2023 | 119.2043* | (49.21; 197.6) | 0.002 |
| | Average (AAPC) | 2007 | 2023 | 6.6747* | (2.77; 10.26) | 0.002799 |
| **Age 75 - 84** | 1 | 2007 | 2018 | 6.4353* | (2.09; 14.48) | 0.005999 |
| | 2 | 2018 | 2021 | -39.1611* | (-50.58; -18.31) | 0.0008 |
| | 3 | 2021 | 2023 | 163.9243* | (67.37; 285.84) | 0.0008 |
| | Average (AAPC) | 2007 | 2023 | 7.3595* | (2.74; 11.68) | 0.0004 |
| **Age 85+** | 1 | 2007 | 2018 | 6.2243* | (2.84; 12.07) | 0.0012 |
| | 2 | 2018 | 2021 | -33.6553* | (-43.79; -16.38) | 0.0016 |
| | 3 | 2021 | 2023 | 94.6846* | (35.77; 159.9) | 0.0016 |
| | Average (AAPC) | 2007 | 2023 | 4.9021* | (1.28; 7.96) | 0.004399 |
| **Associated meningitis** | 1 | 2007 | 2018 | 1.245 | (-2.62; 8.42) | 0.375925 |
| | 2 | 2018 | 2021 | -33.9496* | (-45; -12.57) | 0.026795 |
| | 3 | 2021 | 2023 | 61.6216* | (4.12; 120.92) | 0.041192 |
| | Average (AAPC) | 2007 | 2023 | -0.9206 | (-5.01; 2.4) | 0.668266 |
| **Associated sepsis** | 1 | 2007 | 2018 | 3.5992* | (0.27; 11.23) | 0.037193 |
| | 2 | 2018 | 2021 | -29.5556* | (-39.47; -11.4) | 0.0024 |
| | 3 | 2021 | 2023 | 114.4920* | (48.22; 187.99) | 0.002 |
| | Average (AAPC) | 2007 | 2023 | 5.5492* | (2.08; 9.11) | 0.004399 |
| **Other associated diseases** | 1 | 2007 | 2017 | 30.7738 | (-5.52; 59.09) | 0.062388 |
| | 2 | 2017 | 2021 | -33.9651 | (-61.57; 82.74) | 0.132374 |
| | 3 | 2021 | 2023 | 147.8325 | (-13.8; 407.5) | 0.119576 |
| | Average (AAPC) | 2007 | 2023 | 19.4094* | (7.44; 27.64) | 0.0004 |
| **Untyped** | 1 | 2007 | 2018 | 5.9336* | (1.26; 33.2) | 0.029194 |
| | 2 | 2018 | 2021 | -26.0347* | (-41.34; -2.45) | 0.024795 |
| | 3 | 2021 | 2023 | 145.7186* | (41.81; 265.63) | 0.008398 |
| | Average (AAPC) | 2007 | 2023 | 10.0166* | (4.55; 15.17) | 0.003599 |
| **Non-vaccinal serotypes** | 1 | 2007 | 2018 | 30.4007 | (-0.77; 51.66) | 0.05159 |
| | 2 | 2018 | 2021 | -46.3746 | (-61.63; 74.82) | 0.173965 |
| | 3 | 2021 | 2023 | 85.0191 | (-25.58; 229.03) | 0.19756 |
| | Average (AAPC) | 2007 | 2023 | 15.3224* | (5.51; 21.65) | 0.0008 |
| **PCV7 serotypes** | 1 | 2007 | 2023 | -12.4412* | (-20.13; -4.23) | 0.003199 |
| | Average (AAPC) | 2007 | 2023 | -12.4412* | (-20.13; -4.23) | 0.003199 |
| **PCV13 serotypes** | 1 | 2007 | 2023 | -7.5683* | (-13.52; -1.24) | 0.019196 |
| | Average (AAPC) | 2007 | 2023 | -7.5683* | (-13.52; -1.24) | 0.019196 |
| **PCV15 serotypes** | 1 | 2007 | 2018 | -1.4339 | (-8.82; 12.82) | 0.877425 |
| | 2 | 2018 | 2021 | -43.3899 | (-58.05; 5.1) | 0.05199 |
| | 3 | 2021 | 2023 | 100.9264 | (-14.54; 232.21) | 0.082783 |
| | Average (AAPC) | 2007 | 2023 | -2.8965 | (-10.14; 2.42) | 0.269946 |

| | | | | | | |
|---|---|---|---|---|---|---|
| **PCV20 serotypes** | 1 | 2007 | 2018 | 2.2231 | (-1.77; 8.69) | 0.228354 |
| | 2 | 2018 | 2021 | -46.7554* | (-56.28; -29.32) | 0.0016 |
| | 3 | 2021 | 2023 | 100.8884* | (31.2; 186.81) | 0.0016 |
| | Average (AAPC) | 2007 | 2023 | -1.5738 | (-5.65; 2.04) | 0.465107 |
| **PPSV23 serotypes** | 1 | 2007 | 2018 | 3.2341 | (-0.62; 9.23) | 0.088382 |
| | 2 | 2018 | 2021 | -45.1793* | (-54.28; -28.19) | 0.007199 |
| | 3 | 2021 | 2023 | 82.4778* | (15.74; 158.38) | 0.017197 |
| | Average (AAPC) | 2007 | 2023 | -1.552 | (-5.76; 1.89) | 0.374725 |
| **PCV21 serotypes** | 1 | 2007 | 2017 | 10.1433* | (3.06; 21) | 0.023195 |
| | 2 | 2017 | 2021 | -37.0687* | (-55.9; -19.87) | 0.033993 |
| | 3 | 2021 | 2023 | 80.8346 | (-5.41; 179.69) | 0.064787 |
| | Average (AAPC) | 2007 | 2023 | 1.8827 | (-4.08; 6.28) | 0.335933 |
| **CFR** | 1 | 2007 | 2023 | 2.1959 | (-0.35; 4.85) | 0.10038 |
| | Average (AAPC) | 2007 | 2023 | 2.1959 | (-0.35; 4.85) | 0.10038 |
| **Vaccination coverage** | 1 | 2012 | 2023 | 8.4152* | (3.54; 13.35) | 0.0004 |
| | Average (AAPC) | 2012 | 2023 | 8.4152* | (3.54; 13.35) | 0.0004 |

Legend: PCV, pneumococcal conjugate vaccine; PPSV, pneumococcal polysaccharide vaccine; CFR, case-fatality rate.
